# Drivers of telemedicine use: comparative evidence from samples of Spanish, Colombian and Bolivian physicians

**DOI:** 10.1186/s13012-014-0128-6

**Published:** 2014-10-08

**Authors:** Francesc Saigí-Rubió, Joan Torrent-Sellens, Ana Jiménez-Zarco

**Affiliations:** Department of Health Science and Internet Interdisciplinary Institute, Open University of Catalonia, Av. Tibidabo 39-43, Barcelona, 08035 Spain; Department of Economics and Business and Internet Interdisciplinary Institute, Open University of Catalonia, Av. Tibidabo 39-43, Barcelona, 08035 Spain

**Keywords:** Telemedicine, Technology acceptance and adoption, Medical profession, Telemedicine diffusion, Information and communication technology

## Abstract

**Background:**

The aim of the study presented in this article is to analyse the determinants of telemedicine use. To that end, the study makes two basic contributions. First, it considers six working hypotheses in the context of technology acceptance models (TAMs). Second, it uses data obtained for three samples of physicians from three different countries (Spain, Colombia and Bolivia). Obtaining and comparing evidence on an international scale allows determinants of telemedicine use to be evaluated across different contexts.

**Methods:**

In Bolivia, the survey was conducted in hospitals and health care centres of the urban and rural districts of the municipality of Sucre, in a population comprising a total of 350 physicians. In Spain, the survey population consisted of medical professionals of all profiles affiliated with health care within the Canary Islands Health Service, comprising a total of 356 physicians. Finally, in Colombia, it was conducted in the Society of Surgery Service at San José Hospital of Bogotá, in a population comprising a total of 184 physicians. Using an extended TAM and survey data from 510 physicians (113 in Spain, 118 in Colombia and 279 in Bolivia), binary logistic regression analysis was performed.

**Results:**

In the three samples, it was found that the physician's level of information and communication technology (ICT) use in his/her personal life was the variable that had the highest explanatory power regarding telemedicine use. In the Spanish sample, the physicians' perceived ease-of-use of ICTs in clinical practice and propensity to innovate were the two other variables that determined telemedicine use, whereas in the Colombian and Bolivian samples, it was the level of optimism about ICTs.

**Conclusion:**

The results facilitated a more complete model that includes personal, usability, and innovatory aspects in the explanation of *Telemedicine use* in Spain, whereas the results for the Latin American samples indicated a more primary model in the explanation of *Telemedicine use*, which was completed by an optimism factor that did not emerge in the Spanish sample.

**Electronic supplementary material:**

The online version of this article (doi:10.1186/s13012-014-0128-6) contains supplementary material, which is available to authorized users.

## Introduction

A range of methodological and disciplinary approaches have been employed in order to understand the drivers of telemedicine adoption. Technology-oriented research has noted that Roger's diffusion of innovations (DOI) theory and Davis's technology acceptance model (TAM) have been successfully used to understand some of the factors that explain the use of information and communication technologies (ICTs) by health care professionals [1],[2]. Information systems and social-oriented research have highlighted that clinical practice may be intimately interconnected to a range of digital devices and forms of information [3]-[5] and to the ways that users use them in their private lives. Organisation-oriented research has shown that the structure of health care organisations, tasks, public policies, incentives and decision-making processes play a major role in explaining how medical professionals overcome barriers to ICT use [6]. Ethical and legal-oriented research has suggested that changes in the nature of the doctor-patient relationship, the status of health informatics, and also the hardware/software providers tend to have an effect on ICT use by medical professionals [7]. Finally, usability-oriented research has shown that compatibility between clinical ICT systems and physicians' tasks, ICT support for information exchange, communication and collaboration in clinical practice and interoperability and reliability also explain the success of ICT use in health care [8].

The incorporation of telemedicine into clinical practice has generated huge expectations as a means of health care cost containment, since it offers the potential to improve the efficiency and effectiveness of health care organisations, as well as the quality of the services provided [9]-[12]. However, its use in day-to-day clinical practice is still limited [13],[14]. There is general consensus that its slow, arduous implementation can be attributed to the lack of definitive scientific evidence supporting its positive impact on clinical practice (improved quality and effectiveness) and economic outcomes (improved cost-benefit) [13],[15]-[22].

In order to understand the effects of telemedicine use on health outcomes, it is crucial to analyse the prior step, that is, to determine what factors explain physicians' telemedicine use. Obtaining and comparing evidence on an international scale represents an important contribution to the literature, in the sense that it will allow the determinants of telemedicine use to be evaluated across national contexts.

This study analyses and compares the determinants of telemedicine use by samples of physicians from three countries. By doing so, the intention is to attain a twofold objective: first, to characterise and develop a typology of physicians according to their ICT use and expectations in their clinical practice and their personal lives and, second, to identify the determinants that foster or hinder the physicians' telemedicine use. In order to attain the latter part of the objective, this study involved a comparative analysis of three samples of physicians from three Ibero-American countries (Spain, Colombia and Bolivia).

### Hypothesis and model

TAM is the theoretical system most widely applied to research into the acceptance of new information technologies in the professional sphere [23]-[29]. In particular, the model has the capacity to robustly explain the intention to use ICTs, taking into account individuals' perceptions of technology: perceived usefulness and perceived ease-of-use [30],[31]. Thus, the first two hypotheses of the study are as follows:

- H1. The perceived usefulness of ICTs in clinical practice explains telemedicine use.
- H2. The perceived ease-of-use of ICTs in clinical practice explains telemedicine use.

Despite its widespread acceptance, this model has a series of limitations that mainly stem from the fact that it does not take the influence of other types of variables into account. Bagozzi [32] and Venkatesh et al. [33] underscored the need to increase the explanatory power of this model by incorporating additional variables. According to Davis, identifying variables like these in TAM can increase the explanatory power of the system users' acceptance [24],[34]. This is particularly important to the development of TAMs in the field of health care. The consideration of variables relating to the individual, to his or her psychological or sociodemographic characteristics, and to his or her exposure to social influence is a standard feature. Consequently, the theoretical model used needs to take into account contributions made by other models such as those:

1. Tending to stress the importance of technology, such as DOI [35]
2. Tending to stress the importance of the user profile and of social influence (subjective norm), such as the theory of reasoned action (TRA) [36] and the theory of planned behaviour (TPB) [37]
3. That have a mixed approach, comprising elements that refer to both the user profile and to technology, such as Parasuraman's theory of technology readiness (TR) [38]

TR considers the need to incorporate user profile elements, in the sense of mental states resulting from facilitators or inhibitors, which together have the potential to determine one's predisposition towards using new technologies. In this respect, the third hypothesis is as follows:

- H3. The user's optimism about technology explains telemedicine use.

Similarly, we must consider that while each person's stance on technology is different, in a group of people, each stance can be situated on a continuum ranging from strongly positive to strongly negative. Thus, peoples' stances, as situated on this continuum, are correlated to their propensity to adopt and use new technology. In this respect, the fourth hypothesis is as follows:

- H4. The user's propensity to innovate explains telemedicine use.

Finally, it should be noted that physicians use ICTs in their professional and personal lives. As an ICT user, the medical professional may use technology with differing degrees of intensity and frequency. It would seem clear that an individual who uses ICTs intensively is more likely to adopt them in his or her clinical practice, hence, the need to incorporate the intensity of ICT use as an explanatory factor of telemedicine use. In this respect, the fifth hypothesis is as follows:

- H5. The ICT user profile of the physician - as an individual, in his or her personal life - explains telemedicine use.

**Figure 1:**
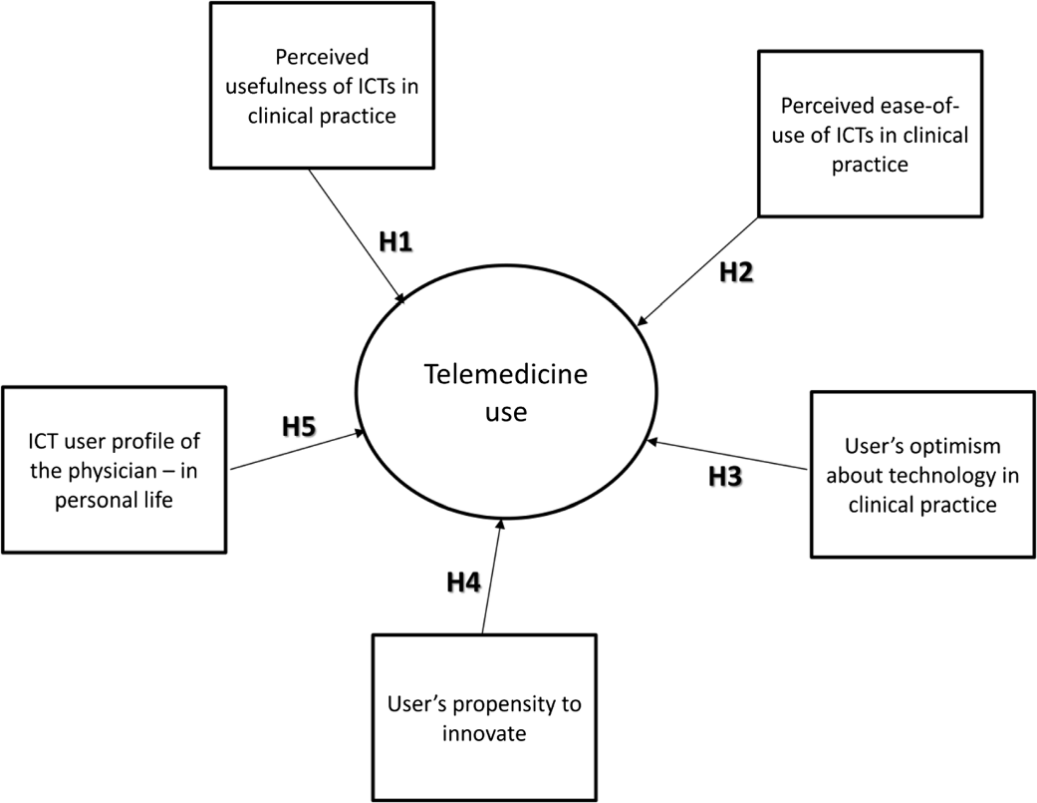
**Drivers of telemedicine use model.**

A summary of the proposed model and hypotheses is shown in Figure 1.

In addition to the determinants identified above, we stress the importance of the geographical, legal, social, cultural and economic contexts of the country or territory [39] to the adoption of ICTs in clinical practice. The digital divide refers to the existence of varying degrees of ICT implementation and adoption in different countries. Economic circumstances clearly influence the quantity and quality of technological equipment and infrastructure. In some sectors, social and cultural aspects also play a key role [11], as do other circumstantial variables such as experience and training [40]. Depending on the country being analysed, the above model can be expected to show significant differences, and this is proposed in the sixth hypothesis:

- H6. The degree of ICT implementation in the country's health care system explains the importance of each of the determinants of the physician's telemedicine use.

### Data collection, empirical methodology and validation

Three samples of physicians from three countries with varying degrees of implementation of ICT use in clinical practice were taken as the reference: Spain, Colombia and Bolivia. The study compares samples of physicians from different territories with varying levels of development according to the economic indicators provided by the IMF for 2012 and 2014 [41]:

1. Spain, a developed country belonging to the European Union, with a GDP of US$1,340.266 billion and GDP per capita of US$30,740 in 2012 according to IMF estimates. According to the official statistics provided by the Spanish Ministry of Health, Social Services and Equality [42], spending on public health care, including long-term care, was €58.466 billion. This represents 71.2% of the country's total health care spending, which is €82.064 billion. As a percentage of GDP, total health care spending in Spain is 8.4%. According to the data provided by the World Bank [43], the mean annual percentage of GDP spent on health care in the 2009–2013 period was 9.3%.
2. Colombia, a developing country with a GDP of US$365.402 billion and a GDP per capita of US$11,284 in 2012. The lack of official statistics for public health care spending in Colombia means that we have taken the figures provided by the World Bank as the reference indicator. That institution states that, for the 2009–2013 period, mean health care spending as a percentage of GDP was 6.8% [43].
3. Bolivia, a country with low level of development and a GDP of US$26.749 billion and a GDP per capita of US$4,996 in 2012.

Table 1 shows the descriptive information for the data collection in each of the three contexts.

**Table 1:** **Described information for the data collection conducted in Spain, Colombia and Bolivia**

	Spain	Colombia	Bolivia
Sampling universe	356 physicians from hospitals, health care services and research centres of the Canary Islands Health Service	184 physicians affiliated to the Society of Surgery Service, San José Hospital of Bogotá	350 physicians from hospitals and health care centres in Sucre
Sample	113	118	279
Interview	Self-administered questionnaire	Self-administered questionnaire	Self-administered questionnaire
Margin of error	7.6% *p* = *q* = 95% confidence level	5.4% *p* = *q* = 95% confidence level	2.7% *p* = *q* = 95% confidence level
Fieldwork	January to June 2012	February to April 2012	August to September 2011

### Instrument design: the CICUS project

The use of a methodology and working tools that have already been validated within an international project is a strength of this work. The Ibero-American Cluster for University Collaboration on Health (CICUS, reference C/030942/10) is an international scientific cooperation project led by the Open University of Catalonia (UOC) in accordance with the criteria set out in the Scientific Research and Inter-University Cooperation Programme (PCI) between Spain and Latin America, funded in full by the Spanish Agency for International Development Cooperation (AECID).

The mission of the CICUS project is to promote and develop innovation, research, technological and academic cooperation programmes in the field of Health Sciences through the collaborative networking of the various centres forming part of it. The CICUS vision is to become a space of excellence that facilitates networking for the creation and sharing of state-of-the-art knowledge on new forms of health care based on the integration of ICTs, which respond to the challenges posed by the socioeconomic changes of the 21st century.

The project began in 2010 with the participation of the UOC (Spain), Simón Bolívar Andean University (UASB, Bolivia) and the Central University of Ecuador (UCE, Ecuador). Performing an analysis of the situation regarding the participating countries' ICT use in the field of health care was one of the first goals set. A qualitative methodology was chosen, and a questionnaire was designed as the data collection instrument.

A review of the literature together with the health care professionals' experience served as the basis to create the study variables and the metrics used in the first version of the instrument. The final questionnaire was organised into four blocks of questions: sociodemographic and professional background; opinions about ICT and Internet use in health care; degree of ICT and Internet use in general and degree of IT-system, ICT and Internet use at work. The instrument was validated following a pre-test. This was done through a pilot study that involved having 48 postgraduate students (all health care professionals) in the master's degree programmes in Public Health and in Clinical Pharmacology offered by UASB's Health Area that completes the measure. This took place for 2 months during the academic year (from 10 May 2011 to 15 June 2011). The instrument was tested and modified in accordance with the suggestions made by the respondents (Additional file 1).

Besides the UOC's Faculty of Health Sciences and the UASB's Health Area, two new organisations took part: the Canary Islands Health Service (SCS) and the Society of Surgery Service, San José Hospital of Bogotá, Colombia. The same questionnaire was used for all three samples, thus ensuring that the variables measured and the metrics used were exactly the same. A preliminary questionnaire was sent for revision to the other two organisations participating in our study (the Canary Islands Health Service (SCS) and the Society of Surgery Service, San José Hospital of Bogotá, Colombia). The purpose of this stage was to finalise the drafting, content and general design of the questionnaire and to test both the instrument and its suitability to the contextual characteristics of each country. Between 9 and 27 January 2012, the questionnaire was sent for revision and adaptation to several health care professionals with academic and practical experience with information systems at the SCS. A similar procedure was implemented in Colombia between 23 January and 7 February 2012. After the instrument had been tested and modified in accordance with the respective experts' suggestions, we proceeded with the study in the three samples of physicians from the three countries in question (Additional files 2 and 3).

The survey was anonymous and optional. Throughout the process, all the necessary procedures were implemented to safeguard the confidentiality of the information and the anonymity of the participants in all study materials. The participants were also informed about the potential restrictions in relation to the confidentiality of the information provided.

### Data collection and populations

In Bolivia, the study was conducted in hospitals and health care centres of the urban and rural districts of the municipality of Sucre. It had 115 health care establishments (87% primary care, 8% secondary care and 5% tertiary care), where a total of 350 physicians practised. The health care professionals were contacted in person between August and September 2011. After explaining the reasons for and aim of the study, potential participants were asked to collaborate by answering the survey, which was completed by hand. Data collection was concluded in mid September, with a total of 279 completed surveys (79.7% response rate). The survey data were then entered manually into an electronic. This was done through the collaboration of postgraduate students on the master's degree programmes in Public Health and in Clinical Pharmacology offered by UASB's Health Area.

In Spain, the study population consisted of medical professionals of all profiles affiliated with the field of health care within the SCS, comprising a total of 356 physicians across health care centres, secondary care hospitals and research centres/universities, who received an invitation to take part in the SCS online survey by e-mail. The fieldwork was carried out between 31 January and 28 June 2012, with a total of 113 completed surveys (31.7% response rate).

Finally, the Society of Surgery Service at San José Hospital of Bogotá had a staff of 184 physicians, who attended to an average of 161,000 patients per year in outpatient services. On 13 February 2012, the medical director of the Society of Surgery Service, San José Hospital of Bogotá, sent an invitation to take part in the online survey by e-mail to the 184 health care professionals. The fieldwork was carried out between 13 February and 30 April 2012, with a total of 118 (64.1% response rate) completed surveys. No mechanism to incentivise the health care professionals' participation was implemented in any of the three cases.

### Study variables

Table 2 shows the variables used in the study.

**Table 2:** **Study variables**

Variable	Definition	Scale
Telemedicine use	ICT use for diagnosing, monitoring and treating patients in situations where those involved are separated by place and/or time [44]	Dichotomous variable. 0 = no; 1 = yes. The variable is additive and considers ICT use for the purposes of contacting national and international professionals of exchanging valid information for diagnosing, monitoring and treating patients and of disseminating research activities with the aim of improving people's health
Ease-of-use of ICTs in clinical practice	Degree to which the physician considers that using ICTs in clinical practice means that less efforts will be required to perform his or her tasks	Categorical variable. 1 = perceived ease-of-use is nil; 2 = very low; 3 = low; 4 = average; 5 = quite high; 6 = high; 7 = very high
Perceived usefulness of ICTs in clinical practice	Degree to which the physician believes that using ICTs in clinical practice improves his or her performance in an activity in the short-term	Metric variable obtained from exploratory factor analysis. The original variables included in the analysis are measured on a 5-point Likert scale. The variables making up the factor are those indicating how the physician perceives that using ICTs enables errors to be controlled when performing an activity or a patient's evolution to be monitored
Optimism	Degree to which the physician considers that using ICTs in clinical practice will enable him or her to obtain benefits or reduce effort in the future	Metric variable obtained from exploratory factor analysis. The original variables included in the analysis are measured on a 5-point Likert scale. The variables making up the factor are those indicating how the physician perceives that using ICTs enables relationships with patients to be improved and better treatments to be recommended
Propensity to innovate	The individual's propensity to innovate in his or her day-to-day work using ICTs	Categorical variable. 5 = the individual totally agrees; 1 = totally disagrees
Level of ICT use	Degree to which the physician uses ICTs outside work	Dichotomous variable. 0 = his or her ICT use is low; 1 = high

The hypothesised multidimensional nature of the variables *Perceived usefulness of ICTs in clinical practice* and *Optimism* suggested that exploratory factor analysis (EFA) should be performed.

EFA is a data dimensionality reduction technique. Starting with the analysis of a set of original variables, it seeks to determine the smallest number of dimensions capable of explaining the maximum amount of information contained in the data [45]. In order to extract the factor dimensions, seven variables were considered in total. Each variable was designed to assess the physicians' perceived benefits of using ICTs in their clinical practice.

As Patil et al*.*[46] suggest, EFA typically requires decisions about three key issues: factor extracting strategy, factor retention criteria and factor rotation. For our three samples, the exploratory analysis was performed by applying the principal axis extraction and Promin rotation methods [47]. In this context, polychoric correlation matrices were used. These are particularly suitable for items with a Likert-type response format [48]. Also, in order to obtain the appropriate number of factors, an approach combining multiple criteria were used: the scree test^a^[49]-[51] and parallel analysis^b^[46],[52]-[54].

The Factor 9.20 programme [55] was used to perform the EFA. This programme allows polychoric correlation matrices to be analysed and procedures such as parallel analysis to be performed. Parallel analysis was performed using the optimal parallel analysis option in Factor 9.20 in which characteristics of the data were taken into account, and the polychoric correlations and random permutations of the answers were utilised [54].

Table 3 shows the variables included in the factor analyses for the three samples of physicians. It also shows the factor scores obtained, the percentage variance explained by each factor, and the component transformation matrix.

**Table 3:** **Main factor analysis results**

	Spain		Colombia		Bolivia	
	Perceived usefulness of ICTs in clinical practice	Optimism	Perceived usefulness of ICTs in clinical practice	Optimism	Perceived usefulness of ICTs in clinical practice	Optimism
Variables						
Looking up medical or health-related information on the Internet improves the doctor-patient relationship		0.577		0.852		0.680
Looking up medical or health-related information on the Internet leads to doubts about the health care professional's knowledge		0.747		0.809		0.705
Looking up medical or health-related information on the Internet improves knowledge of the patient and facilitates his or her treatment		0.765		0.809		0.735
The existence of computerised data allows the evolution of the patient's clinical status to be viewed	0.946		0.772		0.788	
I am in favour of creating a single computerised medical history for each patient, which can be accessed by any health care professional regardless of the centre where the patient is attended	0.943		0.785		0.844	
With excessive ICT use, there is greater control of errors	0.862		0.639		0.783	
In most cases, computerisation and ICT use in the field of health care minimise bureaucracy and have a major impact on improved clinical practice	0.703		0.770		0.605	
% variance explained	0.57788	0.15195	0.15521	0.47019	0.42973	0.14720
Eigenvalue	4.0451	1.06362	1.08644	3.29133	3.00810	1.03041
Cronbach's alpha	0.811	0.874	0.891	0.877		
Correlation matrix	Correlated variables, values higher than 0.5		Correlated variables, values higher than 0.5		Correlated variables, values higher than 0.5	
Determinant of the matrix	0.001992139587910		0.156882043355530		0.318942324331276	
KMO index	0.90176		0.88039		0.73541	
Bartlett's statistic	645.7***		410.8 ***		514.1***	
Measurement of sample adequacy	Coefficients between 0.62 and 0.80		Coefficients between 0.62 and 0.80		Coefficients between 0.62 and 0.80	
Component correlation matrix	1	2	1	2	1	2
1	1.000	0.646	1.000	0.525	1.000	1.000
2	0.646	1.000	0.525	1.000	0.400	1.000

***Significant at 99% confidence level.

In order to confirm the technique's goodness of fit for the three samples, various statistical tests were performed in accordance with the proposals by Bilodeu and Brenner [56], Fabrigar et al. [45] and Nunnally and Bernstein [57]. The analysis of the sample correlation matrix for the three analyses performed showed that the variables were highly intercorrelated. In every case, the correlation coefficient values were higher than 0.5. Likewise, the Bartlett's test of sphericity values were high, indicating that the null hypothesis of multivariate normality was rejected in every case, thus confirming that the variables were highly interrelated and appropriate for factor analysis. Also, the Kaiser-Meyer-Olkin (KMO) coefficient indices obtained for the three samples were 0.90176 (Spain), 0.88039 (Colombia) and 0.73541 (Bolivia)^c^. That confirms the adequacy of the matrix for factor analysis.

**Figure 2:**
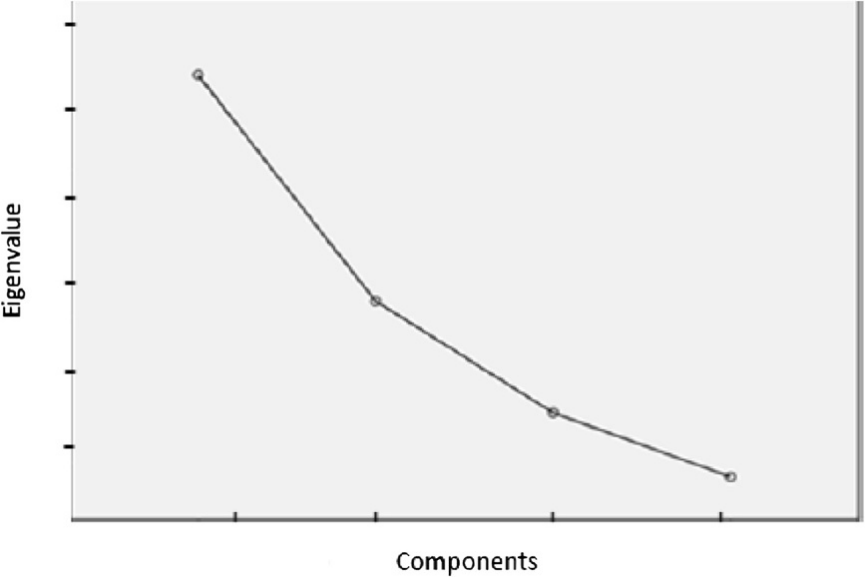
**Sedimentation graph of the Spanish sample.**

**Figure 3:**
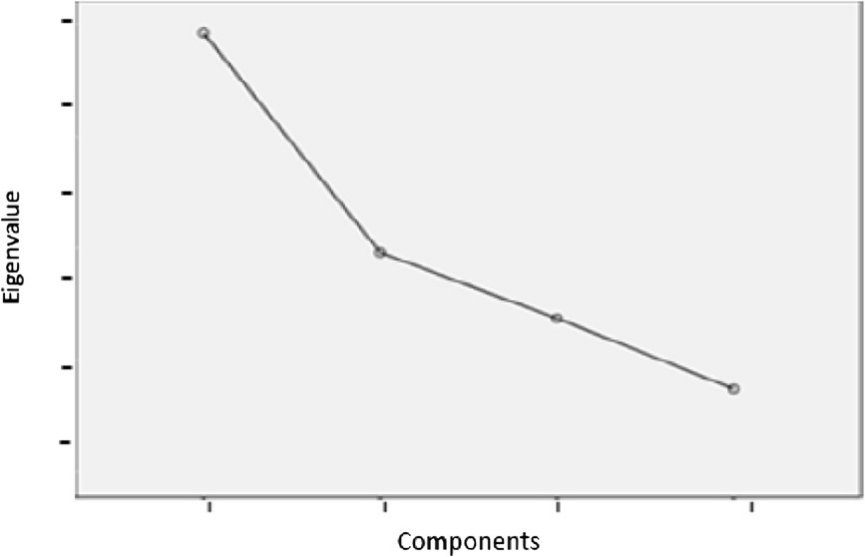
**Sedimentation graph of the Colombian sample.**

**Figure 4:**
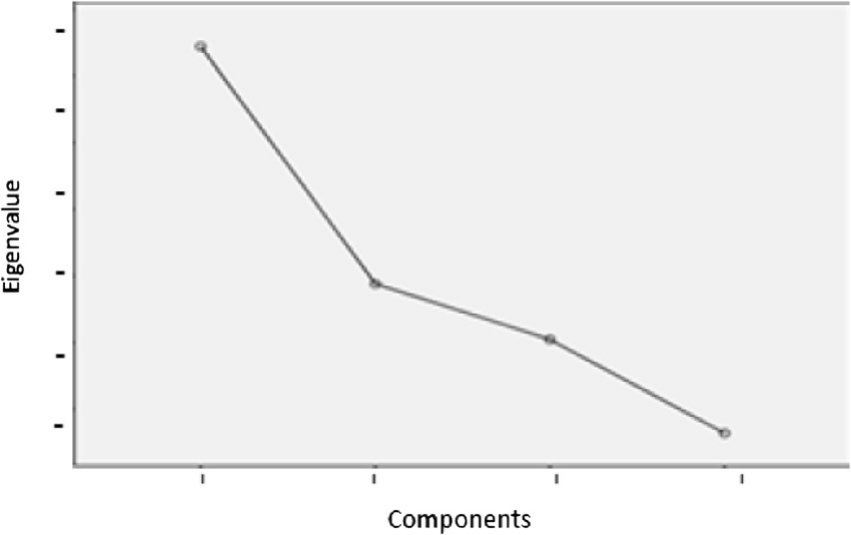
**Sedimentation graph of the Bolivian sample.**

As shown in Figures 2, 3 and 4, the scree test yielded two-factor solutions in all of the three analyses. Parallel analysis also yielded these two-factor structures.

After determining the number of factor to be extracted, the Promin correlated factors rotation method was used [47]. This method of oblique rotation tends to yield the most parsimonious solution.

As shown in Table 3, each of the three different analyses performed enabled two factors to be obtained. Taking into account the seven variables considered in the analysis, the factors were identified with the constructs *Optimism* and *Perceived usefulness of ICTs in clinical practice*. In two of the three analyses identified, the factor called perceived usefulness had a higher eigenvalue and explained a higher percentage of total variance. In particular, for the Spanish sample, the total variance explained by these two factors was 72.9%. The two factors present a high correlation (*r* = 0.646). Regarding the Colombian sample, the total variance explained by the factors was 62.6%. The two factors show a moderate correlation (*r* = 0.525). Finally, for the Bolivian sample, the two factors explained 57.69% of the total variance. The two factors also show a moderate correlation (*r* = 0.400).

Finally, it should be noted that using the above-mentioned factors as explanatory variables of the proposed theoretical model meant that the reliability of the metric scales had to be calculated. As shown in Table 3, the Cronbach's alpha coefficient value obtained for each of the constructs was high. It was higher than 0.70 in every case. According to Nunnally and Bernstein [57], this indicator must have values higher than 0.7 in general and 0.6 in the case of new scales. Thus, it is possible to assume that the scales used were reliable. Additionally, the content and construct scales' discriminant, convergent and nomological validity were also addressed. With regard to the content, the scales were developed following a major review of the literature.

### Preliminary evidence obtained

#### Physician's profile

Regarding the physician's profile, in the three samples analysed, it was found that the majority was male (almost 60%). In the sample of Spanish physicians, almost 40% were over 50 years of age and had been in post for a considerable length of time (44.4% had over 20 years' experience). In comparison to this profile, the physicians in the Colombian sample were younger; 59.9% were between 40 and 50 years of age. Finally, the physicians in the Bolivian sample were younger still; 58% were under 40 years of age (see Table 4).

**Table 4:** **Descriptive analysis of the physicians' characteristics**

		Spain	Colombia	Bolivia
Gender	Male	67.3	60.2	40.5
	Female	32.7	39.8	59.5
Age	<40 years	14.2	59.0	50.6
	40–50 years	30.1	27.3	33.3
	50–60 years	46.0	8.6	12.9
	>60 years	9.7	5.1	3.2
Workplace	Health care centre's central services	8.3		
	Research centre University	0.9	23.7	
	Tertiary care hospital	46.9	37.3	13.3
	Secondary care hospital	8.0	13.7	56.6
	Health care centre	35.8	31.3	30.1
Occupation	Administrative post	18.5	26.2	8.9
	Non-administrative post	81.4	73.7	91.0

Irrespective of origin, in more than half of the cases analysed, it was found that the medical professional had a permanent contract or was even a career civil servant and worked mainly in secondary and tertiary care hospitals.

Regarding the physician's relationship with technology, and his or her perceptions and personal and professional use of it, significant differences between the three samples were found. These differences appeared to stem from the individual's profile and the country where he or she worked.

Table 5 shows the results of analyses of variance (ANOVAs), where *Gender*, *Age* and the *Post* occupied by the physician in the health care institution were included as independent variables, and the physician's degree of *Optimism* and *Propensity to innovate*, *Perceived usefulness of ICTs in clinical practice* and perceived *Ease-of-use of ICTs in clinical practice*, *Level of ICT use* and *Telemedicine use* were dependent variables. In order to facilitate data analysis and sample comparison, the *Age* and *Post* variables were dichotomised. Consequently, it was considered that young professionals were under 45 years of age, and the level of responsibility associated with a medical professional's post was higher when he or she performed managerial, directorship or operational group manager functions.

**Table 5:** **Descriptive statistics (ANOVA analysis)**

	Descriptive statistics														
	Gender	***N***	Mean	Standard deviation	***F***	Age	***N***	Mean	Standard deviation	***F***	Post	***N***	Mean	Standard deviation	***F***
Spain															
Optimism	Female	37	−0.0535	1.11965	-	<45	33	0.0526	1.04442	-	No responsibility	92	0.0516	0.97009	NS
	Male	76	0.0279	0.94657		>45	80	−0.0212	0.99450		Responsibility	21	−0.2270	1.14416	
	Total	113	0.0000	1.00471		Total	113	0.0000	1.00471		Total	113	0.0000	1.00471	
Perceived usefulness of ICTs in clinical practice	Female	37	0.0306	1.02422	**	<45	33	0.1881	0.82224	-	No responsibility	92	0.0786	0.95412	*
	Male	76	−0.0161	0.95961		>45	80	−0.0758	1.06505		Responsibility	21	0.3463	1.16729	
	Total	113	−0.0001	1.00481		Total	113	−0.0001	1.00481		Total	113	−0.0001	1.00481	
Ease-of-use of ICTs in clinical practice	Female	37	4.4054	0.55073	**-**	<45	33	4.3636	0.82228	-	No responsibility	92	4.4130	0.68182	NS
	Male	76	4.4605	0.70125		>45	80	4.4750	0.57313		Responsibility	21	4.5714	0.50709	
	Total	113	4.4425	0.65381		Total	113	4.4425	0.65381		Total	113	4.4425	0.65381	
Propensity to innovate	Female	37	4.49	0.559	**-**	<45	33	4.18	0.635	*	No responsibility	92	4.33	0.681	NS
	Male	76	4.30	0.731		>45	80	4.44	0.691		Responsibility	21	4.52	0.680	
	Total	113	4.36	0.682		Total	113	4.36	0.682		Total	113	4.36	0.682	
Telemedicine use	Female	37	0.4054	0.49774	**	<45	33	0.4242	0.50189	-	No responsibility	92	0.5109	0.50262	NS
	Male	76	0.6053	0.49204		>45	80	0.5875	0.49539		Responsibility	21	0.6667	0.48305	
	Total	113	0.5398	0.50063		Total	113	0.5398	0.50063		Total	113	0.5398	0.50063	
Level of ICT use	Female	37	0.7568	0.43496	**	<45	33	0.8485	0.36411	-	No responsibility	92	0.8370	0.37143	NS
	Male	76	0.9079	0.29110		>45	80	0.8625	0.34655		Responsibility	21	0.9524	0.21822	
	Total	113	0.8584	0.35019		Total	113	0.8584	0.35019		Total	113	0.8584	0.35019	
Colombia															
Optimism	Female	47	−0.0718	0.79464	**-**	<45	88	−0.0201	1.05199	-	No responsibility	87	−0.0168	1.04077	NS
	Male	71	0.0475	1.12464		>45	30	0.0592	0.86231		Responsibility	31	0.0473	0.90804	
	Total	118	0.0000	1.00420		Total	118	0.0000	1.000420		Total	118	0.0000	1.00420	
Perceived usefulness of ICTs in clinical practice	Female	47	−0.0906	0.93006	**-**	<45	88	0.0681	0.99543	-	No responsibility	87	0.0102	0.98079	NS
	Male	71	0.0599	1.05260		>45	30	−0.1997	1.02010		Responsibility	31	−0.0287	1.08359	
	Total	118	0.0000	1.00421		Total	118	0.0000	1.00421		Total	118	0.0000	1.00421	
Ease-of-use of ICTs in clinical practice	Female	47	1.13	1.583	**-**	<45	88	0.92	1.432	-	No responsibility	87	0.80	1.337	NS
	Male	71	0.73	1.230		>45	30	0.80	1.270		Responsibility	31	1.13	1.522	
	Total	118	0.89	1.388		Total	118	0.89	1.388		Total	118	0.89	1.388	
Propensity to innovate	Female	47	2.98	1.011	**-**	<45	88	3.18	1.023	-	No responsibility	87	3.18	1.006	NS
	Male	71	3.27	1.055		>45	30	3.07	1.112		Responsibility	31	3.06	1.153	
	Total	118	3.15	1.043		Total	118	3.15	1.043		Total	118	3.15	1.043	
Telemedicine use	Female	47	0.2128	0.41369	**	<45	88	0.3636	0.48380	-	No responsibility	87	0.2874	0.45515	*
	Male	71	0.4085	0.49505		>45	30	0.2333	0.43018		Responsibility	31	0.4516	0.50588	
	Total	118	0.3305	0.47240		Total	118	0.3305	0.47240		Total	118	0.3305	0.47240	
Level of ICT use	Female	47	0.5532	0.50254	**	<45	88	0.6932	0.46382	-	No responsibility	87	0.7011	0.46041	NS
	Male	71	0.7606	0.42978		>45	30	0.6333	0.49013		Responsibility	31	0.6129	0.49514	
	Total	118	0.6780	0.46925		Total	118	0.6780	0.46925		Total	118	0.6780	0.46925	
Bolivia															
Optimism	Female	113	−0.0412	1.16294	**-**	<45	184	−0.0411	1.06828	-	No responsibility	254	0.0254	0.98879	NS
	Male	166	0.0280	0.87805		>45	95	0.0795	0.85832		Responsibility	25	−0.2587	1.11443	
	Total	279	0.0000	1.00181		Total	279	0.0000	1.00181		Total	279	0.0000	1.00181	
Perceived usefulness of ICTs in clinical practice	Female	113	−0.1198	0.94670	**	<45	184	−0.0282	1.04199	-	No responsibility	254	0.0230	0.99880	NS
	Male	166	0.0815	1.07059		>45	95	0.0544	0.92169		Responsibility	25	−0.2344	1.02209	
	Total	279	0.0000	1.00175		Total	279	0.0000	1.00175		Total	279	0.0000	1.00175	
Ease-of-use of ICTs in clinical practice	Female	113	0.44	0.499	**-**	<45	184	0.46	0.500	-	No responsibility	254	0.46	0.499	**
	Male	166	0.50	0.502		>45	95	0.51	0.503		Responsibility	25	0.68	0.476	
	Total	279	0.48	0.500		Total	279	0.48	0.500		Total	279	0.48	0.500	
Propensity to innovate	Female	113	4.21	0.700	**-**	<45	184	4.22	0.714	-	No responsibility	254	4.24	0.650	NS
	Male	166	4.24	0.662		>45	95	4.25	0.601		Responsibility	25	4.08	0.909	
	Total	279	4.23	0.677		Total	279	4.23	0.677		Total	279	4.23	0.677	
Telemedicine use	Female	113	0.2566	0.43872	***	<45	184	0.3750	0.48544	-	No responsibility	254	0.3543	0.47925	***
	Male	166	0.4639	0.50020		>45	95	0.3895	0.49022		Responsibility	25	0.6400	0.48990	
	Total	279	0.3799	0.48624		Total	279	0.3799	0.48624		Total	279	0.3799	0.48624	
Level of ICT use	Female	113	0.9558	0.20656	**-**	<45	184	0.9457	0.22732	-	No responsibility	254	0.9528	0.21258	NS
	Male	166	0.9458	0.22713		>45	95	0.9579	0.20189		Responsibility	25	0.9200	0.27689	
	Total	279	0.9498	0.21871		Total	279	0.9498	0.21871		Total	279	0.9498	0.21871	

*NS* not significant, ***significant at 99% confidence level, **significant at 95% confidence level, *significant at 90% confidence level.

Regarding the sample of Spanish physicians, *Gender* was found to have an influence on *Perceived usefulness of ICTs in clinical practice*, *Level of ICT use* in personal life and *Telemedicine use*. In particular, men had a higher *Level of ICT use* in their personal lives and more *Telemedicine use* in their clinical practice. In contrast, women had a greater *Perceived usefulness of ICTs in clinical practice*. In every case, the mean value for the group and each sub-group was considered moderate. Also in every case, the relationship between the variables was significant at the 95% confidence level.

On the other hand, the medical professionals over 45 years of age had a greater *Propensity to innovate*, whereas those occupying posts of responsibility had a greater *Perceived usefulness of ICTs in clinical practice*. In both cases, the value of the variables was significant at the 90% confidence level. Regarding the variable *Post*, it should be noted that the two groups differed considerably in size - one was almost five times bigger than the other - so the mean value of the variable for the total sample had a strong bias towards the group of professionals not occupying posts of responsibility.

In the sample of Colombian physicians, it was found that men also had a higher *Level of ICT use* in their personal lives and more *Telemedicine use* in their clinical practice. In both cases, the value of the variables was moderate, being significant at the 95% confidence level. However, medical professionals occupying posts of responsibility had more *Telemedicine use* in their clinical practice, in a population where the number of medical professionals not occupying posts of responsibility was three times higher than those who did. The value of the variables was marginally significant at the 90% confidence level. Taking into account the ages of the individuals in the sample, no significant differences between the mean values of the variables were found.

Finally, in the sample of Bolivian physicians, once again, it was found that men had more *Telemedicine use* and a greater *Perceived usefulness of ICTs in clinical practice*. In both cases, the value of the variables was moderate, being significant at the 99% and 95% confidence levels, respectively. No significant differences were found as regards *Age* in a population where the number of professionals under 45 years of age was two times higher than those over 45. However, significant differences were found as regards the posts they occupied. In a population where the proportion of medical professionals occupying posts of responsibility - such as chief or general manager of medical staff - was 1 to 10, medical professionals occupying posts of responsibility had more *Telemedicine use* and a greater perceived *Ease-of-use of ICTs in clinical practice*. Once again, the value of the variables in both cases was moderate, being significant at the 99% and 95% confidence levels, respectively.

#### Drivers of telemedicine adoption

In order to test the hypotheses proposed above, a binary logistic regression analysis was performed. Table 6 shows the results for the three samples. The goodness of fit of the three samples was high, as confirmed by the values and levels of significance reached by the Chi-square statistics and the Hosmer-Lemeshow test. Moreover, the values of Nagelkerke's statistic indicated that the three samples had explanatory power. The value of this statistic was 27.5% for the Spanish sample, 16.1% for the Colombian sample and 19.7% for the Bolivian sample.

**Table 6:** **Relationships between the explanatory variables and telemedicine use for the three samples of physicians**

	***B***	S.E.	Wald	***Df***	Significant	Exp(B)
Spain						
Perceived usefulness of ICTS in clinical practice	0.200	0.445	0.202	1	0.653	1.222
Ease-of-use of ICTS in clinical practice	0.667	0.364	3.354	1	0.067	1.948
Optimism	−0.498	0.437	1.302	1	0.254	0.607
Propensity to innovate	0.724	0.373	3.769	1	0.052	2.062
Level of ICT use	2.661	1.077	6.103	1	0.013	14.316
Constant	0.724	0.373	3.769	1	0.052	2.062
Chi-square, 24.859; significant, 0.000						
Hosmer-Lemeshow test, 19.273; significant, 0.018						
Nagelkerke R Square, 0.275						
Colombia						
Perceived usefulness of ICTS in clinical practice	−0.192	0.253	0.575	1	0.448	0.826
Ease-of-use of ICTS in clinical practice	0.108	0.182	0.347	1	0.556	1.113
Optimism	0.485	0.269	3.254	1	0.071	1.624
Propensity to innovate	0.361	0.232	2.423	1	0.120	1.435
Level of ICT use	1.212	0.511	5.626	1	0.018	3.360
Constant	−2.878	0.951	9.163	1	0.002	0.056
Chi-square, 14.489; significant, 0.013						
Hosmer-Lemeshow test, 14.564; significant, 0.045						
Nagelkerke R Square, 0.161						
Bolivia						
Perceived usefulness of ICTS in clinical practice	0.131	0.141	0.866	1	0.352	1.140
Ease-of-use of ICTS in clinical practice	0.321	0.256	1.571	1	0.210	1.379
Optimism	0.484	0.150	10.371	1	0.001	1.622
Propensity to innovate	0.033	0.189	0.031	1	0.861	1.034
Level of ICT use	2.040	1.049	3.780	1	0.052	7.689
Constant	-2.224	1.375	2.618	1	0.106	0.108
Chi-square, 20.704; significant, 0.000						
Hosmer-Lemeshow test, 14.705; significant, 0.072						
Nagelkerke R Square, 0.197						

The Spanish sample had a high goodness of fit, explaining more than 27% of the dependent variable's variance. Of the independent variables analysed, only *Level of ICT use*, *Propensity to innovate* and *Ease-of-use of ICTs in clinical practice* had a significant effect. In particular, the health care professional's *Level of ICT use* outside work was the variable that had a higher explanatory power. In order of importance, it was followed by the variables *Propensity to innovate* and *Ease-of-use of ICTs in clinical practice*. In both cases, the value of the variables was moderate, being significant at the 95% and 90% confidence levels, respectively. Taking into account these results, it is possible to conclude that hypotheses H2, H4 and H5 were confirmed for the Spanish sample.

Regarding the Colombian sample, the goodness of fit was high, albeit lower than the previous case. Of the independent variables analysed, only the professional's *Level of ICT use* and *Optimism* about the effects of ICTs on clinical practice were significant. In particular, in the same way as in the previous case, *Level of ICT use* was the variable that had a higher explanatory power. At some distance, it was followed by the variable *Optimism* with a beta value of 0.485, being significant at the 90% confidence level. Taking into account these results, it is possible to conclude that hypotheses H3 and H5 were confirmed for the sample of Colombian health care professionals.

Finally, the Bolivian sample had a lower goodness of fit and a lower explanatory power of the dependent variable. Of the independent variables analysed, only *Level of ICT use* and *Optimism* had a higher explanatory power and level of significance within it. The variable *Level of ICT use* had the greatest weight, with a very high beta coefficient, being significant at the 95% confidence level. *Optimism* was second in order of importance, with a relatively low explanatory power, being significant at 99%. Taking into account these results, it is possible to conclude that hypotheses H3 and H5 were confirmed for the Bolivian sample.

A comparative analysis of the three samples showed that there were differences in the importance of the explanatory variables considered, thus confirming hypothesis H6.

In summary, Table 7 shows the accepted and rejected hypotheses for each of the samples.

**Table 7:** **Accepted and rejected hypotheses for the Spanish, Colombian and Bolivian samples**

Hypotheses	Spain	Colombia	Bolivia
H1. The perceived usefulness of ICTs in clinical practice explains telemedicine use	Rejected	Rejected	Rejected
H2. The perceived ease-of-use of ICTs in clinical practice explains telemedicine use	Accepted	Rejected	Rejected
H3. The user's optimism about technology explains telemedicine use	Rejected	Accepted	Accepted
H4. The user's propensity to innovate explains telemedicine use	Accepted	Rejected	Rejected
H5. The ICT user profile of the physician - as an individual, in his or her personal life - explains telemedicine use	Accepted	Accepted	Accepted
H6. The degree of ICT implementation in the country's health care system explains the importance of each of the determinants of the physician's telemedicine use	Accepted	Accepted	Accepted

## Discussion

Research into the uses of technology in health care usually distinguishes between the analysis of the determinants of use (ex-ante research) and the analysis of the outcomes of use (ex-post). While most studies have focused on the outcomes of technology use, it should be noted that ex-ante analysis is important for several reasons. First, it provides evidence of the drivers and barriers explaining technology use (or not). Second, understanding the factors that explain use means that it may be possible to influence the outcomes of such use.

In this sense, the aim of this study was to explain the determinants of telemedicine use by samples of physicians in three countries: Spain, Colombia and Bolivia. Obtaining and comparing evidence on an international scale from an ex-ante analysis determining which factors explain physicians' telemedicine use allow the common and distinct aspects of its use to be identified. To that end, a theoretical model based on a modified TAM was used as the analysis tool. Qualitative methodology was used to design a questionnaire as the data collection instrument. The questionnaire was sent to the professionals working for the health care organisations taking part in the study. In the three samples, the physician's *Level of ICT use* in his or her personal life had the highest explanatory power for *Telemedicine use*. This is understandable because telemedicine can be considered an advanced ICT use. Thus, if a physician uses ICTs in his or her personal life, he or she will be more likely to use them in his or her clinical practice too. However, despite the fact that it was the most important variable in all three samples, the value of its coefficient varied considerably. The highest coefficient was found in the Spanish sample, followed by the Bolivian sample and the Colombian sample.

In the case of the Spanish sample, *Telemedicine use* was also determined by the physicians' perceived *Propensity to innovate* and *Ease-of-use of ICTs in clinical practice*. The results showed a clear relationship between *Level of ICT use* in personal life, perceived *Ease-of-use of ICTs in clinical practice* and *Propensity to innovate* in the explanation of *Telemedicine use* by physicians in Spain. The combination of these three factors indicated a more complete model that contemplated personal, usability and innovatory aspects in the explanation of *Telemedicine use* in Spain.

Regarding the Colombian and Bolivian samples, the variables explaining *Telemedicine use* were *Level of ICT use* in personal life, as mentioned previously, and the level of *Optimism* about ICTs.

Unlike the behaviour observed in the sample of Spanish physicians, the results for the Latin American samples indicated a more primary model in the explanation of *Telemedicine use*. Despite the fact that *Level of ICT use* in personal life was also the main explanatory component, *Telemedicine use* in Colombia and Bolivia was not explained by either *Ease-of-use of ICTs in clinical practice* or *Propensity to innovate*. The explanatory model was completed by an *Optimism* factor that did not emerge in the Spanish sample.

The most plausible explanation is the well-known problems in health care in Latin America, where a variety of factors limit access to high-quality, timely health care, such as the scarcity of human resources, medication, equipment, infrastructure and cultural and geographical accessibility to health care services [58]. And Colombia and Bolivia are no exception to this. In a context plagued by deficiencies such as these, it would seem logical to think that physicians should be more optimistic about telemedicine's potential to resolve those deficiencies. In Spain, however, where there is a greater implementation of ICTs in health care, optimism about telemedicine's potential was no less important, ceding ground to ease of use of ICTs in clinical practice and the physicians' propensity to innovate.

The study presented in this article has several limitations, particularly the empirical approach taken, the lack of a prospective study design and the variables and restrictions imposed on the analysis. However, the availability of three samples of physicians from three different countries provided an excellent opportunity to analyse the determinants of telemedicine use. In this respect, and bearing in mind the importance of this type of analysis to health care organisations, the availability of more data on other groups of physicians to widen the comparison, a time series, better indicators and new analyses relating the determinants of telemedicine use to its outcomes would suggest that new approaches could be taken. In addition, the study's sample limitations are of particular note. Although the comparison made is relevant, increasing the number of observations to improve the representativeness of the samples, for both the set of physicians and for the health care systems, will be a future line of study in our research programme.

## Conclusions

Two substantive conclusions can be drawn from this work. First of all, the considerable value that the variable physician's *Level of ICT use* - as an individual, in his or her personal life - has in the main explanatory power on *Telemedicine use*. Second, our findings suggest that *Telemedicine use* can also be determined by other factors of health care services (such as the lack of human resources, infrastructure, equipment, medication and cultural and geographical accessibility) and the level of ICT implementation in the field of health care, conferring different models in the explanation of its use.

These results support for the need for a dynamic approach to the design of telemedicine use, especially when it targets a variety of end users from different countries and health care systems. Hence, the importance of conducting studies prior to using telemedicine and attempting to identify which of the above-mentioned variables could exert an influence and how. Research is confronted with the challenge of producing such evidence, a prerequisite for the generalised adoption of telemedicine [59]. In this respect, it is reasonable to assume that the variations in barriers to technology adoption have more to do with local factors than technology infrastructure development. This scenario poses a considerable challenge for the formulation of public policies and strategies by countries and states, where decisions regarding can affect ultimate adoption and implementation of technological innovations. The potential to reduce limitations to health care access and improve the health sector's efficiency can therefore be considered a twofold positive aspect of incorporating telemedicine use into health care.

### Endnotes

^a^Cattell's scree test is a widely known approach for determining the number of factors. In this procedure, the eigenvalues of the correlation matrix are computed and then plotted in order of descending values. Thus, the method offers a visual exploration of a graphical representation of the eigenvalues.

^b^Parallel analysis is a method based on the generation of random variables to determine the number of factors to retain. Thus, the method compares the observed eigenvalues extracted from the correlation matrix to be analysed with those obtained from uncorrelated normal variables. Zwick and Velicer [53] show that among the different factor retention criteria that can be used, parallel analysis is the most accurate, showing the least variability and sensitive to different factors.

^c^Bartlett's test of sphericity and the KMO test are two measures of Appropriateness of Factor Analysis. Bartlett's test of sphericity is used to test the null hypothesis that the variables in the population correlation matrix are uncorrelated. In other words, this analysis tests if the correlation matrix is an identity matrix, i.e. all diagonal elements are 1 and all off-diagonal elements are 0, implying that all of the variables are uncorrelated. If the significant value for this test is less than our alpha level, we reject the null hypothesis that the population matrix is an identity matrix. The significant value for this analysis leads us to reject the null hypothesis and conclude that there are correlations in the data set that are appropriate for factor analysis. On the other hand, the KMO index is used to compare the magnitudes of partial correlation coefficients; the lower its value is, the higher the partial correlation coefficients' values are, and consequently, the less appropriate it is to perform EFA [56]. According to the proposal made by those authors, coefficient values equal to or higher than 0.5 are acceptable*.*

## Additional files

## Electronic supplementary material

Additional file 1: Final questionnaire used in the sample of Bolivian physicians.(DOC 354 KB)

Additional file 2: Final questionnaire used in the sample of Spanish physicians.(DOC 198 KB)

Additional file 3: Final questionnaire used in the sample of Colombian physicians.(DOC 204 KB)

Below are the links to the authors’ original submitted files for images.

Authors’ original file for figure 1

Authors’ original file for figure 2

Authors’ original file for figure 3

Authors’ original file for figure 4
