# Supplementary material for: Drivers of telemedicine use: comparative evidence from samples of Spanish, Colombian and Bolivian physicians
Source: Implement Sci. 2014 Oct 8;9:128. doi: 10.1186/s13012-014-0128-6 (PMC4195871; doi:10.1186/s13012-014-0128-6)
Supplement: Supplementary file 1 — Additional file 1: Final questionnaire used in the sample of Bolivian physicians.(DOC 354 KB) [file 13012_2014_128_MOESM1_ESM.doc]

**Ibero-american Cluster for University Cooperation on Health (CICUS)**

**Simón Bolívar Andean University (UASB)-Central University of Ecuador (UCE)-Open University of Catalonia (UOC)**

**with the support of the Spanish Ministry of Foreign Affairs and Cooperation (MAEC)-Agency for International Development Cooperation (AECID)**

**SURVEY OBJECTIVES**

1. To describe the use of information and communication technologies (ICTs) in the field of healthcare in the participating countries, by age / gender / position / specialty / centre.
2. To describe and analyse the perceptions, attitudes and needs of healthcare professionals in relation to ICT use.
3. To identify the situation in which the consortium’s universities find themselves, in order to determine priority training needs.
4. To formulate a set of recommendations to enable progress to be made on the development of programmes for academic and technological cooperation, and research and innovation in healthcare through ICT use, in accordance with the specific needs of each constituent country.

**THE SURVEY HAS FOUR SECTIONS:**

SECTION 1. Personal and professional background data

SECTION 2. Opinion of ICT and Internet use in the field of healthcare

SECTION 3. Level of ICT and Internet use in general

SECTION 4. Level of IT-system, ICT and Internet use at work

**Welcome to the survey on ICT use in the healthcare organisation where you work**

We would be very grateful if you could help us by filling in the *ICT use in the field of healthcare* questionnaire, which forms part of the research conducted within the Ibero-American Cluster for University Cooperation on Health (CICUS) project, whose participating universities are UASB, UCE and UOC. This project analyses the process of interaction between ICTs and healthcare in Bolivia and Ecuador.

Any personal data extracted from this survey will be treated confidentially and used in aggregate form only, as a statistical basis for the final report and any other scientific publications that may be produced.

We hope that the results of the survey will contribute to a more effective and participatory form of management, and that is why we are asking you to take part.

Thank you.

It will take approximately 15 minutes of your time to answer this survey.

SECTION 1. PERSONAL AND PROFESSIONAL BACKGROUND DATA

| **1.** | Year of birth | | | |  | |  | | |
| --- | --- | --- | --- | --- | --- | --- | --- | --- | --- |
|  | | | | | | | | | |
| **2.** | Gender | |  | | | | | | |
|  |  | | Male | X | |  | | | |
|  |  | | Female | X | |  | | | |
|  | | | | | | | | | |
| **3.** | Please indicate your professional category: | | | | | | | | |
|  |  | Specialist physician | | | | | | X |  |
|  |  | Resident physician | | | | | | X |  |
|  |  | General physician | | | | | | X |  |
|  |  | Graduate nurse | | | | | | X |  |
|  |  | Auxiliary nurse | | | | | | X |  |
|  |  | Other non-medical healthcare staff member | | | | | | X |  |
|  |  | Administrative and/or managerial staff member[[1]](#footnote-2) | | | | | | X |  |
|  |  | Teaching staff member | | | | | | X |  |
|  |  | Other non-healthcare staff member | | | | | | X |  |
|  |  | Other [Specify] | | | |  | | | |
|  | | | | | | | | | |
| **4.** | The organisation where you do your main work is… | | | | | | | | |
|  |  | A primary care hospital (or health centre) | | | | | | X |  |
|  |  | A secondary care hospital | | | | | | X |  |
|  |  | A tertiary or specialised care hospital | | | | | | X |  |
|  |  | A service provision company[[2]](#footnote-3) | | | | | | X |  |
|  |  | A research centre - University | | | | | | X |  |
|  |  | Health Ministry, Departmental Health Service, Municipal Health Service | | | | | | X |  |
|  |  | Other [Specify] | | | |  | | | |
|  | | | | | | | | | |

| **5.** | What position do you hold in the organisation where you do your main work? | | | | | | | |
| --- | --- | --- | --- | --- | --- | --- | --- | --- |
|  |  | Non-managerial medical staff member (physician, nurse, dental surgeon, or similar) | | X |  | | | |
|  |  | Coordinator - Supervisor | | X |  | | | |
|  |  | Head of nursing / Head of administration | | X |  | | | |
|  |  | Director | | X |  | | | |
|  |  | Manager | | X |  | | | |
|  |  | Other [Specify] |  | | | | | |
|  | | | | | | | | |
| **6.** | In the last year, have you received any of the following types of training? | | | | | | | |
|  |  |  | | In-house: face-to-face | | In-house: online | External: face-to-face | External: online |
|  |  | University education (qualifications: bachelor’s degree, diploma, master’s degree, doctorate) | | X | | X | X | X |
|  |  | Other training connected with your professional field (other qualifications) | | X | | X | X | X |
|  |  | Software training at user level | | X | | X | X | X |
|  | | | | | | | | |

**SECTION 2. OPINION OF ICT AND INTERNET USE IN THE FIELD OF HEALTHCARE**

| **7.** | Do you think that looking up medical or health-related information on the Internet… | | | | | | |
| --- | --- | --- | --- | --- | --- | --- | --- |
|  |  |  | Totally agree | Agree | Neither agree nor disagree | Disagree | Totally disagree |
|  |  | improves the healthcare professional-patient relationship? | X | X | X | X | X |
|  |  | leads to doubts about the healthcare professional’s knowledge? | X | X | X | X | X |
|  |  | improves the patient’s knowledge and facilitates his or her treatment? | X | X | X | X | X |
|  | | | | | | | |
| **8.** | Please indicate your level of agreement with the following statements: | | | | | | |
|  |  |  | Totally agree | Agree | Neither agree nor disagree | Disagree | Totally disagree |
|  |  | The existence of computerised data that allow the evolution of the patient’s clinical status to be viewed is very useful for clinical practice. | X | X | X | X | X |
|  |  | I am in favour of creating a single computerised medical history for each patient, which can be accessed by any healthcare professional regardless of the centre where the patient is attended. | X | X | X | X | X |
|  |  | With excessive ICT use, there is greater control of errors. | X | X | X | X | X |
|  |  | My professional practice suggests to me that it is as important to innovate on the services’ and the institution’s organisational matters as it is to invest in new practices through the use of ICTs. | X | X | X | X | X |
|  |  | In most cases, computerisation and ICT use in the field of healthcare lead to more bureaucracy and have a minimal impact on improved clinical practice. | X | X | X | X | X |
|  | | | | | | | |

**SECTION 3. Level of ICT and Internet use in general**

| **9.** | Please indicate which technologies or tools you use at work, in your private practice and/or in your personal life. | | | | | | | |
| --- | --- | --- | --- | --- | --- | --- | --- | --- |
|  |  | |  | At work | Private practice | Personal life | I do not have this technology | |
|  |  | | Mobile phone with Internet access (WAP, GPRS, 3G, etc.) | X | X | X | X |  |
|  |  | | Desktop computer | X | X | X | X |  |
|  |  | | Laptop / Tablet | X | X | X | X |  |
|  |  | | Electronic organiser (PDA, Palm, etc.) | X | X | X | X |  |
|  |  | | Blackberry (or other similar mobile device) | X | X | X | X |  |
|  | | | | | | | | |
| **10.** | | How often do use the Internet to do the following activities? | | | | | | |
|  | |  |  | Very often | Often | Rarely | Never | I do not use it, but would like to |
|  | |  | General searches in your specialist field. | X | X | X | X | X |
|  | |  | Searches for national scientific articles. | X | X | X | X | X |
|  | |  | Searches for international scientific articles. | X | X | X | X | X |
|  | |  | Contact with other national healthcare professionals. | X | X | X | X | X |
|  | |  | Contact with other international healthcare professionals. | X | X | X | X | X |
|  | |  | Dissemination of your own works. | X | X | X | X | X |
|  | |  | Contact with users (to provide information or support). | X | X | X | X | X |
| **11.** | | How often do you do the following actions? | | | | | | |
|  | |  |  | Very often | Often | Rarely | Never | I do not use it, but would like to |
|  | |  | Participate in websites where there are discussion groups in your specialist field, helping to clarify doubts. | X | X | X | X | X |
|  | |  | Use a discussion list or search health or medicine-related websites when you have doubts about treatments or diagnoses. | X | X | X | X | X |
|  | |  | Participate as an advisor on websites where there are patient groups. | X | X | X | X | X |
|  | | | | | | | | |

**SECTION 4. LEVEL of IT-system, ICT and Internet use at work**

| **12.** | | Do you use, or would you use if you had the choice, any of the following options in your professional practice in the healthcare organisation where you work? | | | | | | | | | | | | | | | | | |
| --- | --- | --- | --- | --- | --- | --- | --- | --- | --- | --- | --- | --- | --- | --- | --- | --- | --- | --- | --- |
|  | |  | |  | | | | | | Yes, I do use it | | I would use it if I had the choice | | No, I would not use it | |  | |  | |
|  | |  | | Electronic reminders of patient visits or tests (Internet, SMS, etc.). | | | | | | X | | X | | X | |  | |  | |
|  | |  | | Electronic delivery of prescriptions (Internet or other means). | | | | | | X | | X | | X | |  | |  | |
|  | |  | | Electronic delivery of medical reports. | | | | | | X | | X | | X | |  | |  | |
|  | |  | | Electronic consultation of the patient’s medical history. | | | | | | X | | X | | X | |  | |  | |
|  | |  | | Electronic consultation of medical reports or test results. | | | | | | X | | X | | X | |  | |  | |
|  | |  | | Consultation of protocols, clinical guidelines, clinical pathways, etc. available online, on the Internet. | | | | | | X | | X | | X | |  | |  | |
|  | | | | | | | | | | | | | | | | | | | |
| **13.** | | How often do you do the following activities at work exclusively in your working hours? | | | | | | | | | | | | | | | | | |
|  | |  | |  | | | | | | Daily | | Weekly | | Monthly | | Occasionally | | Never | |
|  | |  | | Send and/or receive e-mails. | | | | | | X | | X | | X | | X | | X | |
|  | |  | | Upload or download files. | | | | | | X | | X | | X | | X | | X | |
|  | |  | | Connect to the Internet. | | | | | | X | | X | | X | | X | | X | |
|  | |  | | Consult electronic medical reports and/or test results. | | | | | | X | | X | | X | | X | | X | |
|  | |  | | Remotely connect to external applications. | | | | | | X | | X | | X | | X | | X | |
|  | |  | | Send and/or receive instant messages (Messenger, Skype, etc.). | | | | | | X | | X | | X | | X | | X | |
|  | | | | | | | | | | | | | | | | | | | |
| **14.** | | If your answer was Occasionally or Never to any of the activities in the previous question, what was the reason for that answer? | | | | | | | | | | | | | | | | | |
|  | | **Please give a reason ONLY for those activities to which you answered Occasionally or Never in the previous question!** | | | | | | | | | | | | | | | | | |
|  | |  | |  | | | I do not find it necessary | I do not have access | I am unfamiliar with the tool | | Other [Specify] | | | | | | | | |
|  | |  | | Send and/or receive e-mails. | | | X | X | X | |  | | | | | | | | |
|  | |  | | Upload or download files. | | | X | X | X | |  | | | | | | | | |
|  | |  | | Connect to the Internet. | | | X | X | X | |  | | | | | | | | |
|  | |  | | Consult electronic medical reports and/or test results. | | | X | X | X | |  | | | | | | | | |
|  | |  | | Remotely connect to external applications. | | | X | X | X | |  | | | | | | | | |
|  | |  | | Send and/or receive instant messages (Messenger, Skype, etc.). | | | X | X | X | |  | | | | | | | | |
|  | | | | | | | | | | | | | | | | | | | |
| **15.** | If we define Telemedicine as ICT use for diagnosing, monitoring and treating patients in situations where those involved are separated by place and/or time, how often do you use Telemedicine in the healthcare organisation where you work exclusively in your working hours? | | | | | | | | | | | | | | | | | |  |
|  |  | | Daily | | X |  | | | | | | | | | | | | |  |
|  |  | | Weekly | | X |  |
|  |  | | Monthly | | X |  |
|  |  | | Occasionally | | X |  |
|  |  | | Never | | X |  |
|  | | | | | | | | | | | | | | | | | | |  |
| **16.** | Regarding ICT use at work, please indicate whether you agree or disagree with the following statements: | | | | | | | | | | | | | | | | | |  |
|  |  | |  | | | | | | | | | | Yes | | No | | DK/NA | |  |
|  |  | | It improves access to information. | | | | | | | | | | X | | X | | X | |  |
|  |  | | It facilitates communication among healthcare professionals. | | | | | | | | | | X | | X | | X | |  |
|  |  | | It slows down the work system. | | | | | | | | | | X | | X | | X | |  |
|  |  | | It increases data reliability and security. | | | | | | | | | | X | | X | | X | |  |
|  |  | | It improves patient and user care. | | | | | | | | | | X | | X | | X | |  |
|  |  | | It provides flexibility and autonomy. | | | | | | | | | | X | | X | | X | |  |
|  |  | | It leads to job losses. | | | | | | | | | | X | | X | | X | |  |
|  |  | | It facilitates collaboration among different centres. | | | | | | | | | | X | | X | | X | |  |
|  |  | | It is a fundamental tool at work. | | | | | | | | | | X | | X | | X | |  |
|  |  | | It requires an additional training effort. | | | | | | | | | | X | | X | | X | |  |
|  |  | | It increases user satisfaction. | | | | | | | | | | X | | X | | X | |  |
|  |  | | It is a waste of time because of the system’s technical problems and errors. | | | | | | | | | | X | | X | | X | |  |
|  |  | | It improves work productivity (more activity). | | | | | | | | | | X | | X | | X | |  |
|  |  | | It is an interference in the healthcare professional-patient relationship. | | | | | | | | | | X | | X | | X | |  |
|  |  | | It makes process control and monitoring complicated. | | | | | | | | | | X | | X | | X | |  |
|  |  | | It increases the competitiveness of teaching hospitals to the detriment of other centres. | | | | | | | | | | X | | X | | X | |  |
|  |  | | It creates dependency on IT. | | | | | | | | | | X | | X | | X | |  |
|  | | | | | | | | | | | | | | | | | | |  |
| **17.** | If you had the opportunity to do an ICT-mediated training course in your specialist field to help you improve your professional practice, would you do it? | | | | | | | | | | | | | | | | | |  |
|  |  | | Yes | | X |  | | | | | | | | | | | | |  |
|  |  | | No | | X |  |
|  |  | | DK/NA | | X |  |
|  | | | | | | | | | | | | | | | | | | |  |
| **18.** | In your opinion, is there any important aspect that has not been dealt with in this survey? If so, please give your comments below. | | | | | | | | | | | | | | | | | |  |
|  |  | | | | | | | | | | | | | | | | | |  |
|  |  |
|  |  |
|  |  |
|  |  |
|  |  |
|  |  |
|  |  |

1. This means a staff member on the payroll [↑](#footnote-ref-2)
2. This refers to laboratories and companies associated with hospitals. [↑](#footnote-ref-3)
