# Supplementary figures and images for: Drivers of telemedicine use: comparative evidence from samples of Spanish, Colombian and Bolivian physicians
Source: Implement Sci. 2014 Oct 8;9:128. doi: 10.1186/s13012-014-0128-6 (PMC4195871; doi:10.1186/s13012-014-0128-6)

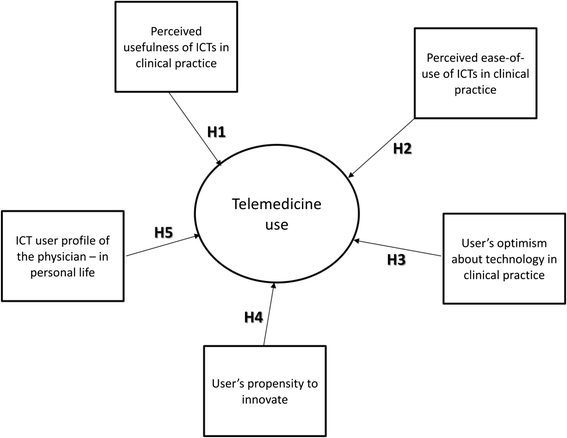

Supplement: Supplementary file 4 — Authors’ original file for figure 1 [file 13012_2014_128_MOESM4_ESM.gif]

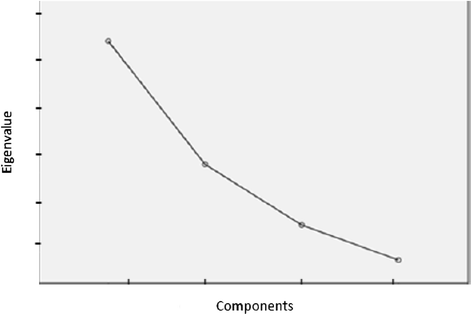

Supplement: Supplementary file 5 — Authors’ original file for figure 2 [file 13012_2014_128_MOESM5_ESM.gif]

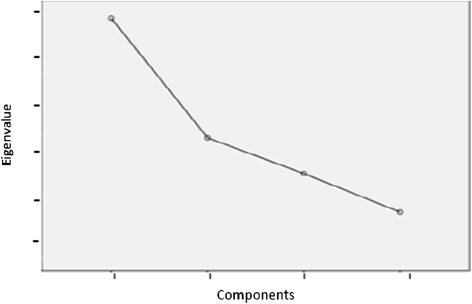

Supplement: Supplementary file 6 — Authors’ original file for figure 3 [file 13012_2014_128_MOESM6_ESM.gif]

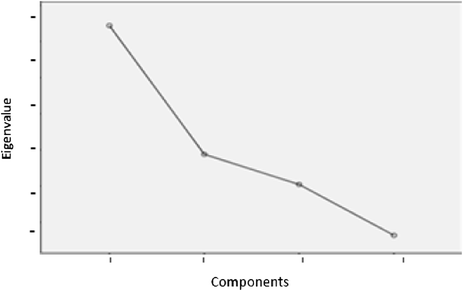

Supplement: Supplementary file 7 — Authors’ original file for figure 4 [file 13012_2014_128_MOESM7_ESM.gif]
